# Association of rare *APOE* missense variants with Alzheimer's disease in the Japanese population

**DOI:** 10.1002/alz70855_098307

**Published:** 2025-12-23

**Authors:** Akinori Miyashita, Ai Obinata, Norikazu Hara, Tamao Tsukie, Mai Hasegawa, Masataka Kikuchi, Kensaku Kasuga, Takeshi Ikeuchi

**Affiliations:** ^1^ Brain Research Institute, Niigata University, Niigata, Niigata, Japan; ^2^ Brain Research Institute, Niigata University, Niigata, Japan

## Abstract

**Background:**

*APOE* is a major susceptibility gene for Alzheimer's disease (AD). Recent studies conducted in Europe and the United States have identified rare missense variants (RMVs) in *APOE* that are significantly associated with AD. However, the presence and role of *APOE* RMVs in East Asians, including the Japanese, and their association with AD and lipid metabolism remain poorly understood.

**Objective:**

To identify *APOE* RMVs in the Japanese population and evaluate their association with AD and lipid metabolism, including low‐density lipoprotein cholesterol levels.

**Subjects:**

*APOE* RMVs were explored in two Japanese cohorts: the Niigata (NIG; 2,589 subjects) cohort and the Tohoku (ToMMo; 3,307 subjects) cohort. A case‐control study was conducted in a Japanese population, including 6,471 individuals with AD and 20,270 control subjects.

**Methods:**

Sanger sequencing and/or whole‐exome sequencing was performed on NIG subjects. We used genotype data from ToMMo. *APOE* RMV frequencies in the Japanese were compared with various ethnic populations. Associations among *APOE* RMV genotypes, AD, and lipoproteins were examined.

**Results:**

Fourteen RMVs were identified (minor allele frequency 0.02 – 0.73%), 10 of which were specifically found in East Asians. Five RMVs previously reported in non‐Japanese populations, including the Christchurch variant (rs121918393: p.R154S [p.R136S]), were not detected in Japanese individuals. Two RMVs (rs140808909 and rs190853081) in complete linkage disequilibrium were associated with protective effects against AD: *P*_Bonferroni_ = 3.63 × 10^‐2^, odds ratio (95% confidence interval) = 0.70 (0.54 ‐ 0.92). No significant differences in cholesterol levels were observed between RMV carriers and non‐carriers.

**Conclusion:**

*APOE* RMVs were identified in the Japanese population, with two variants showing potential protective effects against AD. Further studies with larger cohorts are needed to validate these findings and to explore the role of *APOE* RMVs in AD pathology and lipid metabolism.

